# Maize transcriptome profiling reveals low temperatures affect photosynthesis during the emergence stage

**DOI:** 10.3389/fpls.2025.1527447

**Published:** 2025-01-28

**Authors:** Manja Božić, Dragana Ignjatović Micić, Violeta Anđelković, Nenad Delić, Ana Nikolić

**Affiliations:** ^1^ Laboratory for Molecular Genetics and Physiology, Research Department, Maize Research Institute Zemun Polje, Belgrade, Serbia; ^2^ Gene Bank, Research Department, Maize Research Institute Zemun Polje, Belgrade, Serbia; ^3^ Maize Breeding Group, Breeding Department, Maize Research Institute Zemun Polje, Belgrade, Serbia

**Keywords:** maize, whole transcriptome profiling, low temperature stress, climate change, photosynthesis

## Abstract

**Introduction:**

Earlier sowing is a promising strategy of ensuring sufficiently high maize yields in the face of negative environmental factors caused by climate change. However, it leads to the low temperature exposure of maize plants during emergence, warranting a better understanding of their response and acclimation to suboptimal temperatures.

**Materials and Methods:**

To achieve this goal, whole transcriptome sequencing was performed on two maize inbred lines – tolerant/susceptible to low temperatures, at the 5-day-old seedling stage. Sampling was performed after 6h and 24h of treatment (10/8°C). The data was filtered, mapped, and the identified mRNAs, lncRNAs, and circRNAs were quantified. Expression patterns of the RNAs, as well as the interactions between them, were analyzed to reveal the ones important for low-temperature response.

**Results and Discussion:**

Genes involved in different steps of photosynthesis were downregulated in both genotypes: *psa, psb, lhc*, and *cab* genes important for photosystem I and II functioning, as well as *rca, prk, rbcx1* genes necessary for the Calvin cycle. The difference in low-temperature tolerance between genotypes appeared to arise from their ability to mitigate damage caused by photoinhibition: *ctpa2, grx, elip, UF3GT* genes showed higher expression in the tolerant genotype. Certain identified lncRNAs also targeted these genes, creating an interaction network induced by the treatment (XLOC_016169-*rca*; XLOC_002167-XLOC_006091-*elip2*). These findings shed light on the potential mechanisms of low-temperature acclimation during emergence and lay the groundwork for subsequent analyses across diverse maize genotypes and developmental stages. As such, it offers valuable guidance for future research directions in the molecular breeding of low-temperature tolerant maize.

## 1 Introduction

Negative environmental factors caused by climate change, such as extreme temperatures, and prolonged drought periods, continue to jeopardize global food security by impacting crop productivity (Pörtner et al., 2022; Benitez-Alfonso et al., 2023). Average temperatures on Earth are rapidly rising: an increase of 2.5-4.5°C is expected by the year 2100 (Wang et al., 2018), and this negatively impacts the plants’ carbon uptake, resulting in decreased crop yield (Wang et al., 2018). Maize is particularly affected since it is a C4 plant. Additionally, maize grown in temperate regions reaches the developmental stages most susceptible to heat and drought – flowering and grain filling (Deryng et al., 2014), during a time of year when these extreme weather events are most likely to occur. On average, a yield loss of 7.4% for every 1°C rise in the temperature is predicted for maize (Zhao et al., 2017). Some studies predicted even higher values: up to 23% in the United States (Xu et al., 2016) or 30% in Mexico (Murray-Tortarolo et al., 2018). Since maize is one of the most important crop species worldwide, increasing the crops’ adaptation to climate change, while ensuring sufficient maize yields to guarantee future food security, becomes extremely urgent (Farooq et al., 2023).

Crop adaptation approaches encompass increasing crops’ resilience to climate change factors (Benitez-Alfonso et al., 2023), but also strategies of avoidance, that include earlier sowing, adoption of later-maturing spring varieties, or adoption of winter cultivars in cooler environments (Liu et al., 2017; Collins and Chenu, 2021; Zhu et al., 2022). Some studies have shown that earlier sowing could positively impact maize yield, if implemented with necessary changes in some traits and management practices (Bassu et al., 2021; Zhu et al., 2022). In temperate areas, early sowing could ensure that flowering and grain-filling stages are completed before the periods of extreme heat and drought. However, as maize development is affected by temperatures lower than 15°C (Silva-Neta et al., 2015), earlier sowing results in the exposure of maize seedlings to suboptimal temperatures in the early development, during the emergence stage (VE). The VE stage is considered to be extremely thermo-sensitive, and low temperature (LT) stress at that stage does affect maize yield (Jiang et al., 2021; Beegum et al., 2023).

The effects of low temperatures on maize have been extensively studied over the years. It is known that temperatures below 10°C cause cellular and tissue injuries, protein denaturation, photosynthesis restriction, and oxidative damage (Frascaroli and Revilla, 2018; Zhou et al., 2022a). LT reduces the germination rate and seedling vigor, and leads to the inhibition of growth and development (Yang et al., 2011; Zhang et al., 2020). However, most studies focus on maize seedlings at later developmental stages, starting from the first leaf development (V1) (Waititu et al., 2021; Zhou et al., 2023; Gao et al., 2024). Data on the LT response in the VE stage is less frequent, but recently, more studies on this stage have been published (Li et al., 2021; Xuhui et al., 2022). Implementing early sowing in maize production requires further research of the response to low temperatures during the VE stage, as well as the uncovering of the possible methods of acclimating to this stress factor.

Evaluating gene expression patterns based on transcriptome sequencing (RNA-seq) data has been an effective way of analyzing the molecular mechanisms behind the LT response in plants and elucidating how they combine to establish tolerance (Dasgupta et al., 2020). RNA-seq methods have shown that genes involved in abiotic stress responses, including transcription factors, signal transduction molecules, and antioxidative enzymes, are induced under LT conditions (Mao et al., 2017; Sowiński et al., 2020). Additionally, non-coding RNAs (ncRNAs), such as long non-coding (lncRNAs) and circular RNAs (circRNAs), are involved in gene regulation in plants (Yu et al., 2019). lncRNAs comprise linear, ncRNAs, longer than 200 nucleotides (nt) (Waseem et al., 2021). These ncRNAs fulfill their roles in regulating gene expression through epigenetic modification, splicing regulation, or interaction with other RNA classes (Wang et al., 2023; Gonzales et al., 2024). The primary way lncRNAs regulate gene expression is through their interaction with microRNAs (miRNAs), as described by the ceRNA hypothesis (Salmena et al., 2011; He et al., 2020). miRNAs are known gene expression inhibitors, that act through RNA silencing of target mRNAs (Zhang et al., 2022). The ceRNA hypothesis states that lncRNAs can influence RNA silencing, as they have the same miRNA binding sites as the target mRNAs. By competing for the miRNAs with the target mRNAs, the lncRNAs can reduce the level of target mRNA inhibition and exert their influence on transcription. Circular RNAs are endogenous, single-stranded, covalently-closed RNA molecules, generated by back-splicing events of precursor mRNAs during post-transcriptional processes (Han et al., 2020). Similar to lncRNAs, circRNAs can act as miRNA sponges (Hansen et al., 2013), interact with proteins and transcription factors (Li et al., 2015b), alter genome structure (Dong et al., 2016), and guide protein translation (Yang et al., 2017). Both lncRNAs and circRNAs have been found to take part in the LT response and acclimation of plants (Biswas et al., 2021; Wang et al., 2024). For example, a novel cold-induced lncRNA, CIL1 was found to regulate the expression of multiple stress-related genes, and affect the plant cold tolerance in Arabidopsis (Liu et al., 2022a). Also, overexpression of the *Vitis vinifera* circATS1, improved cold tolerance in Arabidopsis (Gao et al., 2019). Screening for LT regulated genes and ncRNAs in the VE stage may help identify hub genes, ncRNAs, and the interaction networks that can be potential targets for breeding tolerant varieties, capable of survival and growth under LT conditions.

The aim of the research study was to examine the immediate (6h) and delayed (24h) responses to low temperatures in the 5-day-old maize seedlings (VE stage) of both the tolerant and sensitive maize genotype. This was carried out by applying RNA-seq technologies and analyzing the expression of genes and ncRNAs (lncRNAs, circRNAs, and miRNAs) involved in this response. The RNA expression profiles under optimal and LT conditions were compared and the interactions between the different RNA classes were analyzed, with the goal of revealing expression patterns and interaction networks important for establishing low-temperature tolerance in the selected developmental stage.

## 2 Materials and methods

### 2.1 Plant material and experimental design

Two maize inbred lines of contrasting tolerance to LT stress, marked as L_T_, the tolerant, and L_S_, the susceptible inbred line, were exposed to low temperatures at the five-day-old (5d-old) seedling stage. The two maize lines were parental components of commercial ZP hybrids, developed in the Maize Research Institute Zemun Polje (MRIZP), and the seeds were obtained from the MRIZP institute. L_S_ belonged to the Lancaster heterotic group, while L_T_ was a semi-dent. The tolerance of the two lines was assessed in a previous experiment (Božić et al., 2024), and the selection was made based on seed vigor, survival rate, radicle and coleoptile length, as well as seedling fresh weight.

Seeds of both inbred lines were sterilized in 10% sodium hypochlorite (commercial bleach) and germinated in the dark, for five days, under optimal conditions (25/20°C – 12/12h period, 75% relative humidity) in a climate chamber (MLR-352H-PE, PHC Europe B.V.). Subsequently, the 5-day-old seedlings were subjected to the LT treatment (T) at 10/8°C for 6 and 24 hours, 12/12h light/dark photoperiod, and 700 µmolm^-2^s^-1^ light intensity. Samples of 30 maize seedlings *per* inbred line were taken after 6h and 24h for total RNA isolation. The sampled tissue was ground in liquid nitrogen and stored at -80°C until further use. Control plants (C) were grown under optimal conditions (25/20°C – 12/12h photoperiod, 700 µmolm^-2^s^-1^ light intensity, 75% relative humidity) in the same period and sampled at identical time points. There were eight samples in total: two samples per treatment condition (C, T), per time point (6h, 24h), and per inbred line (L_S_, L_T_).

### 2.2 RNA extraction, library preparation, and sequencing

Total RNA was extracted from ≈100 mg of frozen tissue per sample using the GeneJet™ RNA Purification kit (Thermo Scientific, USA) and purified by applying Ambion^®^ DNA-free™ DNase I (Invitrogen, USA), according to the manufacturer’s instructions. Total RNA concentrations were first determined using the NanoPhotometer^®^ spectrophotometer (IMPLEN, USA), while the RNA quality and contamination were screened through agarose gel electrophoresis. After the preliminary check, the 2100 Bioanalyzer and RNA Nano 6000 Assay Kit (Agilent^®^, CA, USA) were used to further determine the RNA integrity and quantity. All samples with RIN above six were chosen for the downstream analysis.

Library preparation and sequencing of the eight samples were carried out on the NovaSeq 6000 (Illumina^®^, USA) at the Novogene Bioinformatics Technology Co. Ltd., in Beijing, China. Firstly, ribosomal RNA, rRNA, was removed from the total RNA (1000 ng) using magnetic beads from the Ribo-Zero Plus rRNA Depletion Kit (Illumina^®^, USA), according to the manufacturer’s instructions. One library per sample was then generated by NEBNext^®^ Ultra™ Directional RNA Library Prep Kit for Illumina^®^ (NEB^®^, USA), following the manufacturer’s recommendations. The library quality was assessed on the Agilent Bioanalyzer 2100. After, the paired-end (PE) 2x150bp sequencing was performed on all eight libraries (L_T_-C-6, L_T_-C-24, L_T_-T-6, L_T_-T-24, L_S_-C-6, L_S_-C-24, L_S_-T-6, and L_S_-T-24).

### 2.3 Data analysis

**Figure 1:**
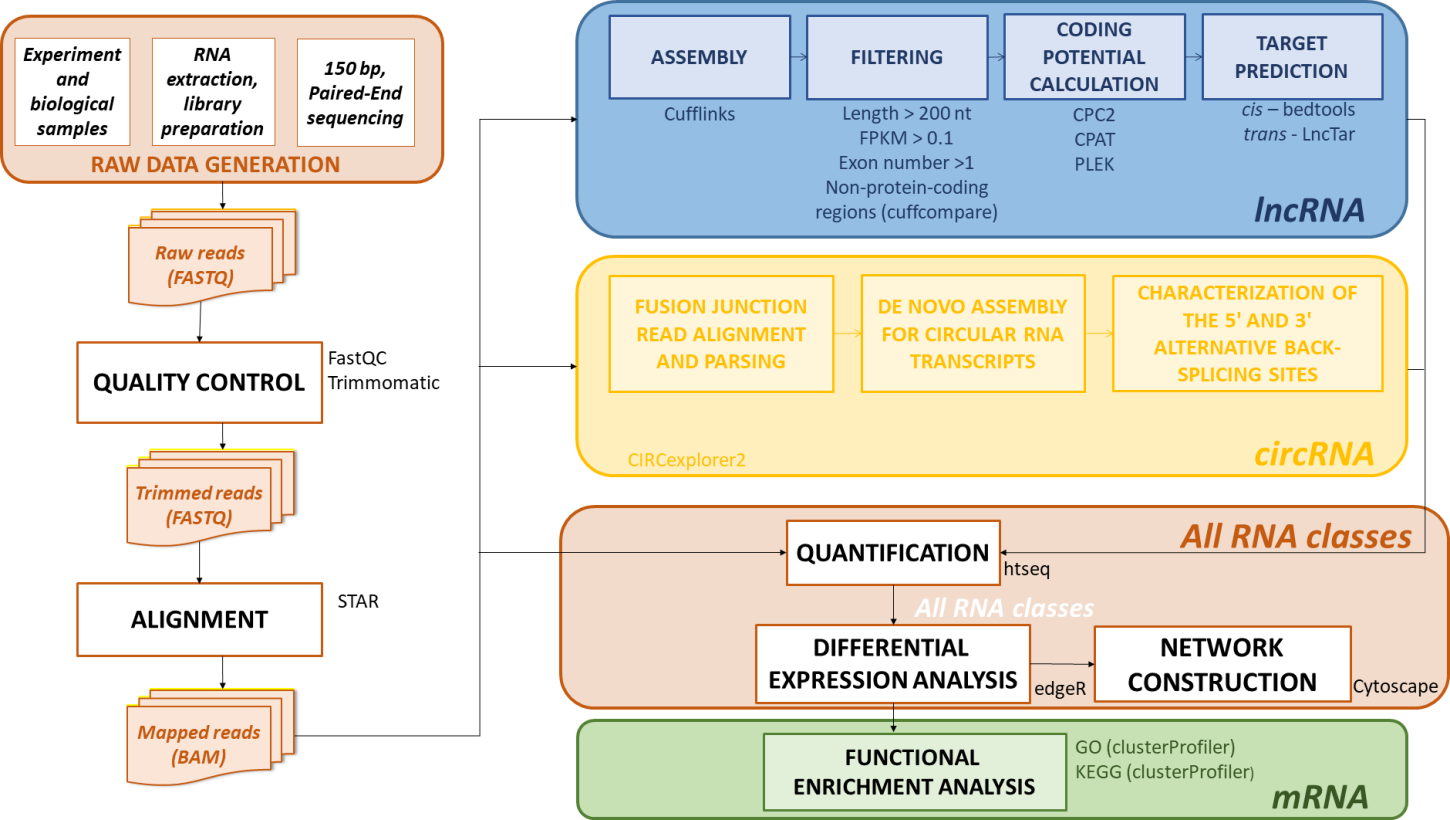
The RNA data analysis pipeline. Raw reads, acquired through 150 bp PE sequencing, were passed through quality control and processed to remove reads containing adapter and poly-N sequences and low quality reads (Phred score > 30, N% < 10%). The trimmed reads were then aligned onto the *Zea mays* B73 NAM 5.0 reference genome. mRNAs could be quantified after mapping, but lncRNAs and circRNAs required additional steps. To identify lncRNAs, the reads were assembled, and filtered (by length, expression level, exon number, and genome position), in addition to assessing the absence of coding potential (CPC2, CPAT, Plek). Additionally, the potential *cis-* and *trans*-targets of the identified lncRNAs were predicted. circRNA detection was accomplished through a custom pipeline in Circexplorer2. After all the coding and non-coding RNAs were identified, the quantification, differential expression (DE) analysis, and network construction were performed. Furthermore, functional enrichment (GO, KEGG) analysis was performed for the identified DE mRNAs. Bioinformatics tools used in each step are also shown next to the box containing the step name.

The raw reads were first checked using FastQC (v0.12.1, Andrews, 2010), and then processed with Trimmomatic (v0.39-2, Bolger et al., 2014). Reads containing adapter and poly-N sequences and low quality reads (Phred score > 30, N% < 10%) were removed. Clean reads were mapped onto the *Zea mays* B73 NAM reference genome version 5.0 (https://plants.ensembl.org/Zea_mays/Info/Index; Accessed March 4, 2024) utilizing STAR (v2.7.11, Dobin et al., 2013) and the corresponding annotation file. The pipelines used for the analysis of all three classes of RNA molecules (mRNAs, lncRNAs, and circRNAs) are shown in **Figure 1**.

#### 2.3.1 mRNA data analysis

Fragments mapped to each gene were quantified with htseq (v2.0.5, Putri et al., 2022). Principal component analysis (PCA) was performed to calculate the distance between samples using *factoextra* (v1.0.7, Kassambara and Mundt, 2020) in R (v4.3.3, R Core Team, 2024). Hierarchical cluster analysis was performed in the *cluster* R package (v2.1.6, Maechler et al., 2023), using normalized read count values and the Euclidian distance method. Differential expression (DE) analysis was performed with edgeR (v4.0, Robinson et al., 2010) to identify significant DE genes between the control and treatment conditions in both L_T_ and L_S_. The transcripts were filtered based on the expression level (minimal counts per sample > 15), library sizes were normalized and the differential expression was calculated. The statistical significance was determined using the negative binomial distribution test (Anders and Huber, 2010; Roberts et al., 2011). The p-value was adjusted according to the Bayesian interpretation (Storey, 2003), and the adjusted value, or the q-value was further used. Genes with the q-value < 0.01 and log2 fold change (FC) > 1 or < -1 between the control and treatment samples were considered differentially expressed.

The Gene Ontology (GO) enrichment analysis of the DEGs was conducted using *clusterProfiler* (v4.10.1, Wu et al., 2021) and biomartr database (Drost and Paszkowski, 2017). Significantly enriched GO terms were determined by the p-value < 0.05 with the Fisher’s exact test and the Bonferroni multi-test adjustment. The same package was used to test the statistical enrichment of the target gene candidates in KEGG metabolic pathways (Kanehisa et al., 2008) by applying the same criteria. Co-expression patterns and gene modules were identified based on the weighted gene co-expression network analysis (WGCNA), by utilizing the WGCNA package in R (v1.72, Langfelder and Horvath, 2008).

#### 2.3.2 lncRNA data analysis

The mapped reads of each sample were assembled after alignment utilizing Cufflinks (v2.2.1, Trapnell et al., 2010) in a reference-based approach. The assembled transcripts were filtered based on their length (> 200 nt), expression level (FPKM > 0.1), and exon number (>1). After that was completed, transcripts that overlap with known protein-coding genes were also filtered out using a Cufflinks function, *cuffcompare*, and only the ones with class codes “u”, “i”, “o” and “x” were kept for further analysis. Then, by applying three different approaches: CPC2 (Kang et al., 2017), CPAT (Wang et al., 2013), and Plek (Li et al., 2014), only the lncRNAs determined to lack any coding potential by all three methods were selected. lncRNA-fragment quantification was performed with htseq and the DE analysis between the control and treatment conditions was carried out utilizing edgeR, adopting the same analysis parameters as those implemented in the mRNA study.

Potential lncRNA *cis-*targets were explored using bedtools (100 kb up- or downstream from the lncRNA coordinates) (v2.31.1, Quinlan and Hall, 2010), while the *trans*-targets were predicted with the LncTar software (ndG < -0.15, Li et al., 2015a). The co-expression patterns of lncRNAs were predicted by applying the WGCNA R package. Additionally, miRNAs expressed under chilling conditions of 5-d old seedlings (Božić et al., 2024) were selected for the miRNA-lncRNA target prediction, performed using psRobot (v1.2, Wu et al., 2012).

#### 2.3.3 circRNA data analysis

circRNA detection and identification of alternative back-splicing sites were accomplished through a custom computational pipeline in CIRCexplorer2 (v2.0.5, Zhang et al., 2016). It included circRNA fusion junction read alignment and parsing; *de novo* assembly for circular RNA transcripts using the Cufflinks reference annotation-based transcript (RABT), and characterization of alternative back-splicing sites, including both the 5’ and 3’ alternative back-splicing events. To quantitate circRNA expression, htseq was utilized using fragments that are mapped to the back-spliced exon-exon junction sites. DE analysis between the treated and control samples was performed in edgeR. microRNA target sites in exons of circRNA loci were identified using psRobot and compared to the miRNAs (Božić et al., 2024).

### 2.4 Construction of the lncRNA/circRNA-miRNA-mRNA network

The results of the DE analysis and target prediction of mRNA, lncRNA, and circRNA, as well as of the previously identified miRNAs (Božić et al., 2024) were utilized in constructing complex miRNA-lncRNA, miRNA-circRNA, and miRNA-mRNA regulatory relationships. Furthermore, the regulatory relationships were then combined into a unique lncRNA/circRNA-miRNA-mRNA regulatory network, established using Cytoscape software (v3.10.2, Shannon et al., 2003).

### 2.5 qRT-PCR validation of the sequencing results

Quantitative real-time PCR (qRT-PCR) was applied to validate the sequencing results. Genes and lncRNAs were chosen for validation based on the FC size and significance, ensuring they are present in most of the sequenced samples. Total RNA was extracted and treated with DNase I, as previously described, from all samples. Revert Aid First Strand cDNA synthesis kit with RNase inhibitor (Thermo Scientific™) was used to synthesize cDNA and the qRT-PCR analysis was carried out using cyclophilin (*cyp*) as the internal reference gene (Lin et al., 2014) and in three biological replicates for each sample for both mRNAs and lncRNAs. PCR reactions were performed on a StepOnePlus™ Real-Time PCR System (Applied Biosystems™, USA) with the HOT FIREPol^®^ EvaGreen^®^ qPCR Mix Plus (ROX) (Solis BioDyne™). The primers used were designed using Primer 3 (v0.4.0) online software (http://bioinfo.ut.ee/primer3-0.4.0) and checked in NCBI Primer-BLAST tool (https://www.ncbi.nlm.nih.gov/tools/primer-blast). The list of the primers is given in the **Supplementary Table S1**. Relative gene expression was calculated according to Livak and Schmittgen (2001) using efficiency correction according to Pfaffl (2003). Student *t*-test was carried out for mean comparison with a significance level at p < 0.05 for qRT-PCR validation results.

## 3 Results

### 3.1 High-throughput sequencing of mRNA-lncRNA-circRNA libraries

In total, 817.8 million raw reads were generated from the sequenced libraries, averaging ≈51.11 million reads per library. After processing the raw reads, 803.2 million high-quality reads remained (on average ≈50.2 million reads). The high-quality reads were then mapped, with the average alignment rate being 93.57% and the unique mapping rate amounting to 78.5%. The detailed information for each sample is listed in **Supplementary Table S2**.

### 3.2 Identification and characterization of LT-responsive mRNAs in maize

**Figure 2:**
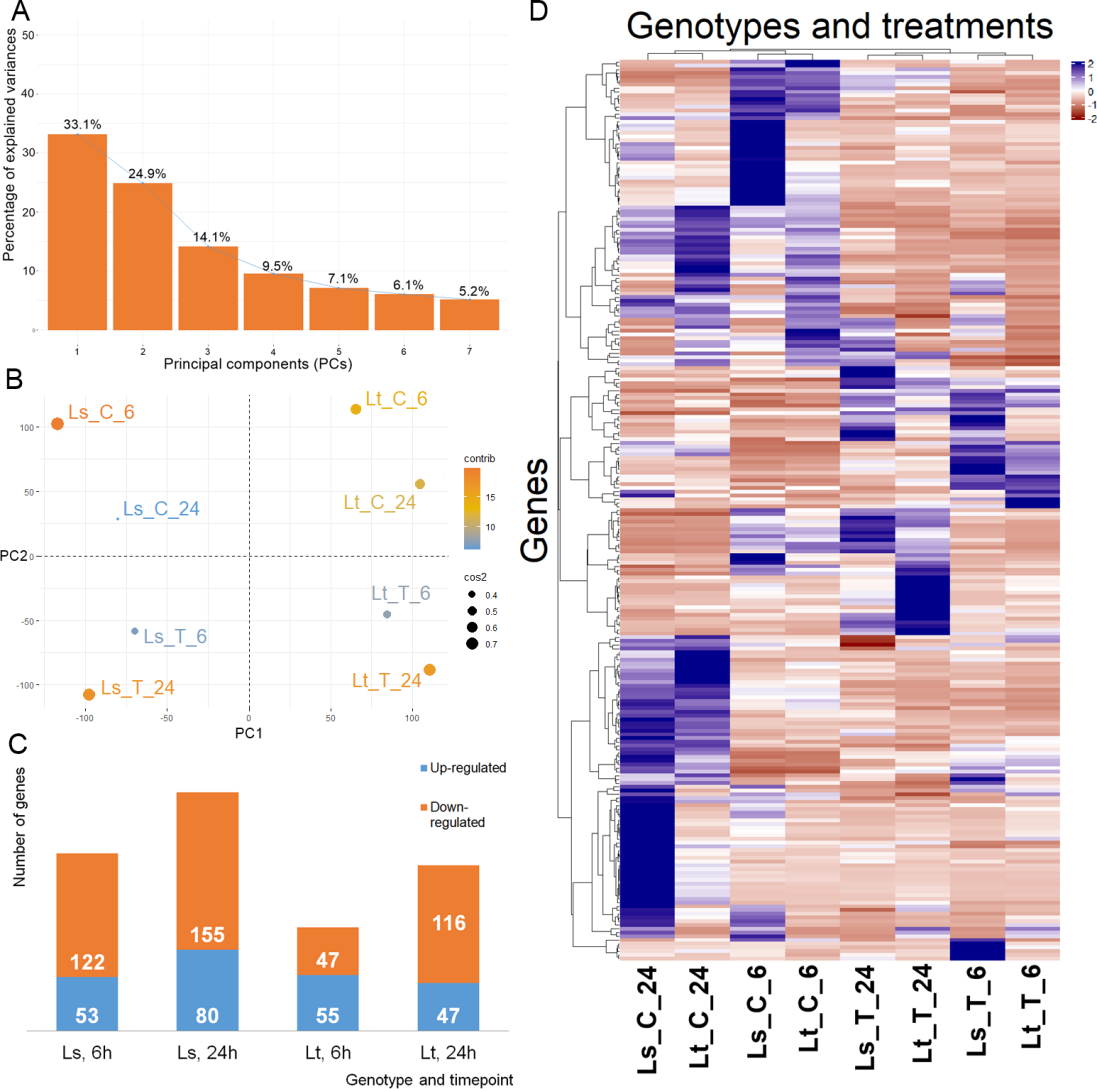
mRNA expression summary. **(A)** Percentage of explained variances of selected principal components (PCs). **(B)** PCA graph of individual libraries, explained by the first two PCs (PC1 and PC2). **(C)** mRNAs differentially expressed between the control and treatment, in the two genotypes (L_S_, L_T_), at the two time points (6h and 24h). Up-regulated mRNAs are shown in blue, while down-regulated are in orange. **(D)** Hierarchical cluster analysis, based on the expression patterns expressed in FPKM of 26,023 mRNAs across the eight libraries.

**Figure 3:**
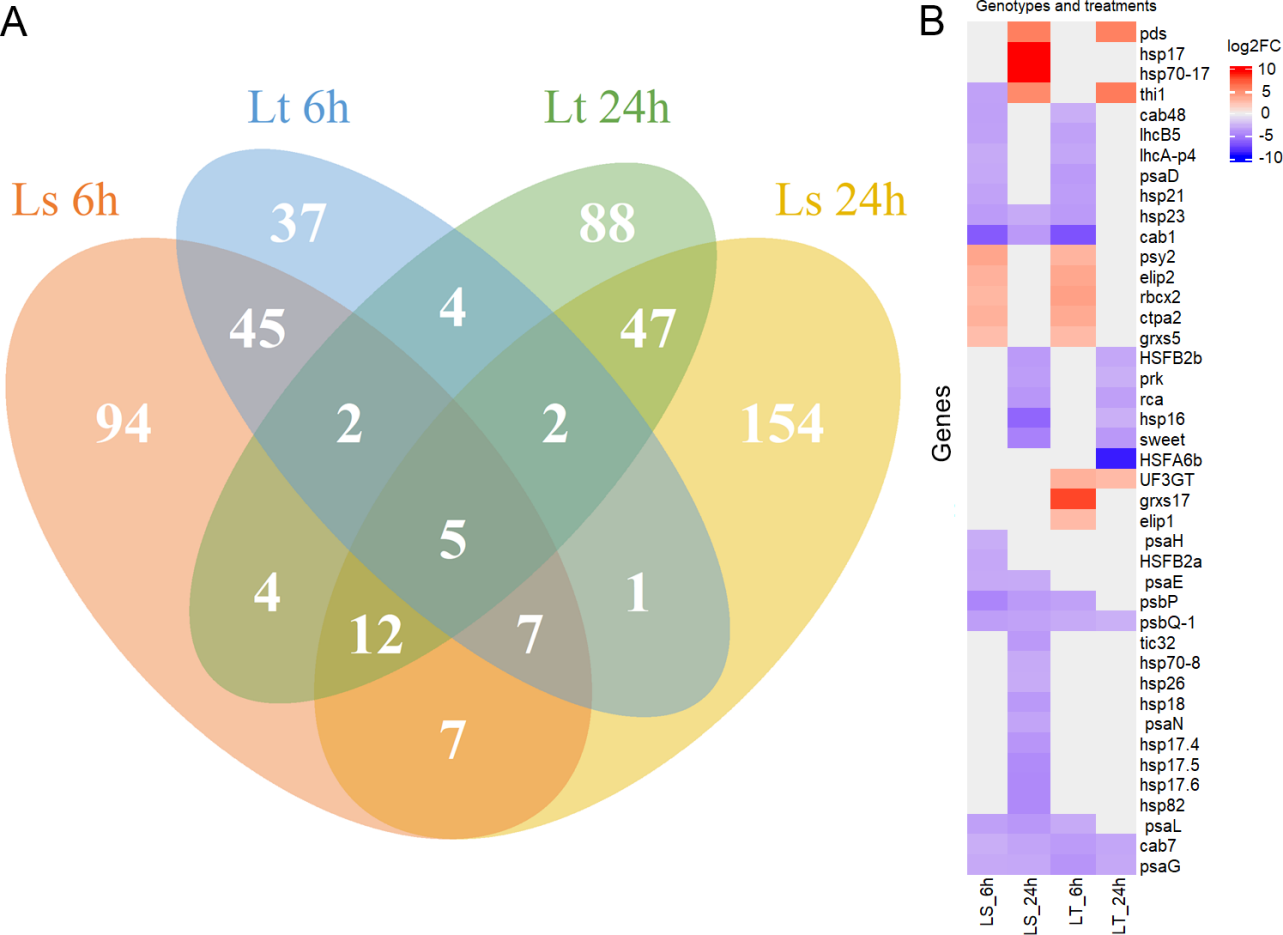
Differentially expressed (DE) genes. **(A)** Venn diagram showing the common and unique differentially expressed (DE) genes for each comparison: L_S_ 6h is shown in orange, L_S_ 24h in yellow, L_T_ 6h in blue, and L_T_ 24h in green. **(B)** Expression profiles of selected DE genes for each comparison (L_S_ 6h, L_S_ 24h, L_T_ 6h, and L_T_ 24h). Fold change is shown as its log 2 value (log2FC), in the range from -10 (blue), over 0 (white), to 10 (red).

To analyze the response of 5-d-old maize seedlings, the expression levels were compared between the control and treatment conditions at both time points (6h, 24h). The mRNA transcripts were quantified, filtered, and FPKM values were calculated. PCA analysis showed that more than 70% of the variability in gene expression abundance between the samples can be explained by the first three principal components (**Figure 2A**). Based on the expression levels, there was a clear separation into four different groups dependent both on the genetic background and experimental conditions (**Figure 2B**). Agglomerative hierarchical clustering analysis additionally confirmed that the samples were clustered firstly based on treatment conditions, and then based on the differences between L_S_ and L_T_ (**Figure 2D**). DE analysis was performed on 26,023 genes, and comparisons were made between the control and treatment conditions. In total, 508 genes were found to be DE (q-value < 0.01, -1 ≤ log2FC ≥ 1): 175 in L_S_ after 6h, and 235 after 24h; while in L_T_ 102 were DE after 6h and 163 after 24h (**Figure 2C**, **Supplementary Table S3**). Five DE genes were common for both genotypes and both time points (Zm00001eb106430, Zm00001eb113780, Zm00001eb161610, Zm00001eb101660, Zm00001eb325410). Common and unique DE genes for each comparison (L_S_ 6h, L_S_ 24h, L_T_ 6h, and L_T_ 24h) are shown in **Figure 3A**.

Most of the DE genes encoded proteins involved in several aspects of photosynthesis. Genes necessary for photosystem I (PSI) assembly – *psa* genes (*psaD, psaE, psaG, psaH, psaL, psaN*), as well as those needed for the assembly of photosystem II (PSII) – *psb* genes (*psbP, psbQ-1*), were down-regulated in both genotypes at either of the time points. Genes important for the stability of the light-harvesting complex (LHC) were also down-regulated – *lhcA-p4, lhcB5, cab1*, *cab7*, *cab48.* Additionally, genes involved in the Calvin cycle and ribulose bisphosphate carboxylase/oxygenase, Rubisco, activity regulation were also found to be DE: *rca*, *rbcx2*, and *prk*. Several genes important for photoinhibition protection were up-regulated: *ctpa2*, *elip1*, *elip2*, *grxs5*, *grxs17*, *psy2*, *thi1*, *UF3GT*, and *pds*. On the other hand, many genes encoding heat shock proteins (HSP) and heat stress transcription factors (HSF) were down-regulated. The hsp/HSF genes, down-regulated after 24h in L_S_, included *hsp16, hsp17.4, hsp17.5, hsp17.6, hsp18, hsp21, hsp23, hsp26, hsp70-8, hsp70-17, hsp82, HSFB2a*, and *HSFB2b*. The only exception was *hsp17*, up-regulated in L_S_ after 24h. On the other hand, only *hsp16, HSFA6b, HSFB2a*, and *HSFB2b* were down-regulated in L_T_ after 24h. Details of the expression profiles of the selected DE genes are shown in **Figure 3B**.

**Figure 4:**
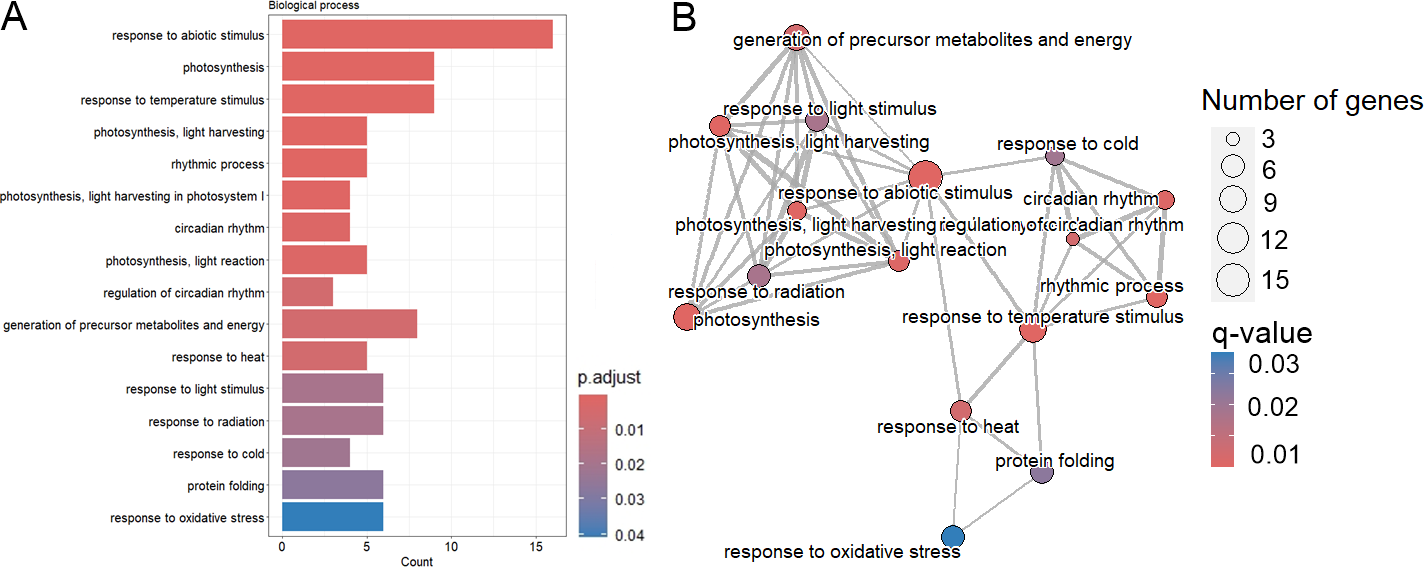
Functional enrichment of differentially expressed mRNAs. **(A)** Bar chart showing the results of over-representation analysis (ORA) for the Biological Process GO class. **(B)** Emap plot showing the results of over-representation analysis (ORA) for the Biological Process GO class.

GO enrichment analysis revealed the DE genes were mainly enriched in two processes: “response to abiotic stimulus” (16 mRNAs) and “photosynthesis” (9 mRNAs). mRNAs enriched in “response to abiotic stimulus” could be further categorized into those enriched in “response to temperature stimulus” (heat, cold), “response to oxidative stress”, but also “response to light stimulus” (**Figure 4**, **Supplementary Table S4**). The KEGG pathway results of the enrichment analyses revealed that the DE mRNAs were mostly enriched in the pathways of “Energy metabolism” (Circadian rhythm) and “Environmental adaptation” (Photosynthesis) (**Supplementary Table S4**).

There were six significant gene clusters (p < 0.05) associated with the LT treatment identified in the WGCNA analysis: three positively and three negatively correlated. Additionally, five gene clusters were significantly associated with the tolerant genotype, L_T_. Among the gene clusters associated with the LT treatment or the tolerant genotype, the genes were filtered based on the module membership and the significance of the expression of the gene in question to the associated trait. The resulting genes and modules are shown in **Supplementary Table S5**. GO enrichment analyses were performed on these genes, showing that genes of three modules associated with the LT treatment were significantly enriched in peptide biosynthetic process and nucleosome organization. On the other hand, genes belonging to modules associated with the tolerant genotype were functionally enriched in response to water deprivation, response to increased salt levels, and nucleosome organization.

### 3.3 Identification and characterization of LT-responsive lncRNAs in maize

**Figure 5:**
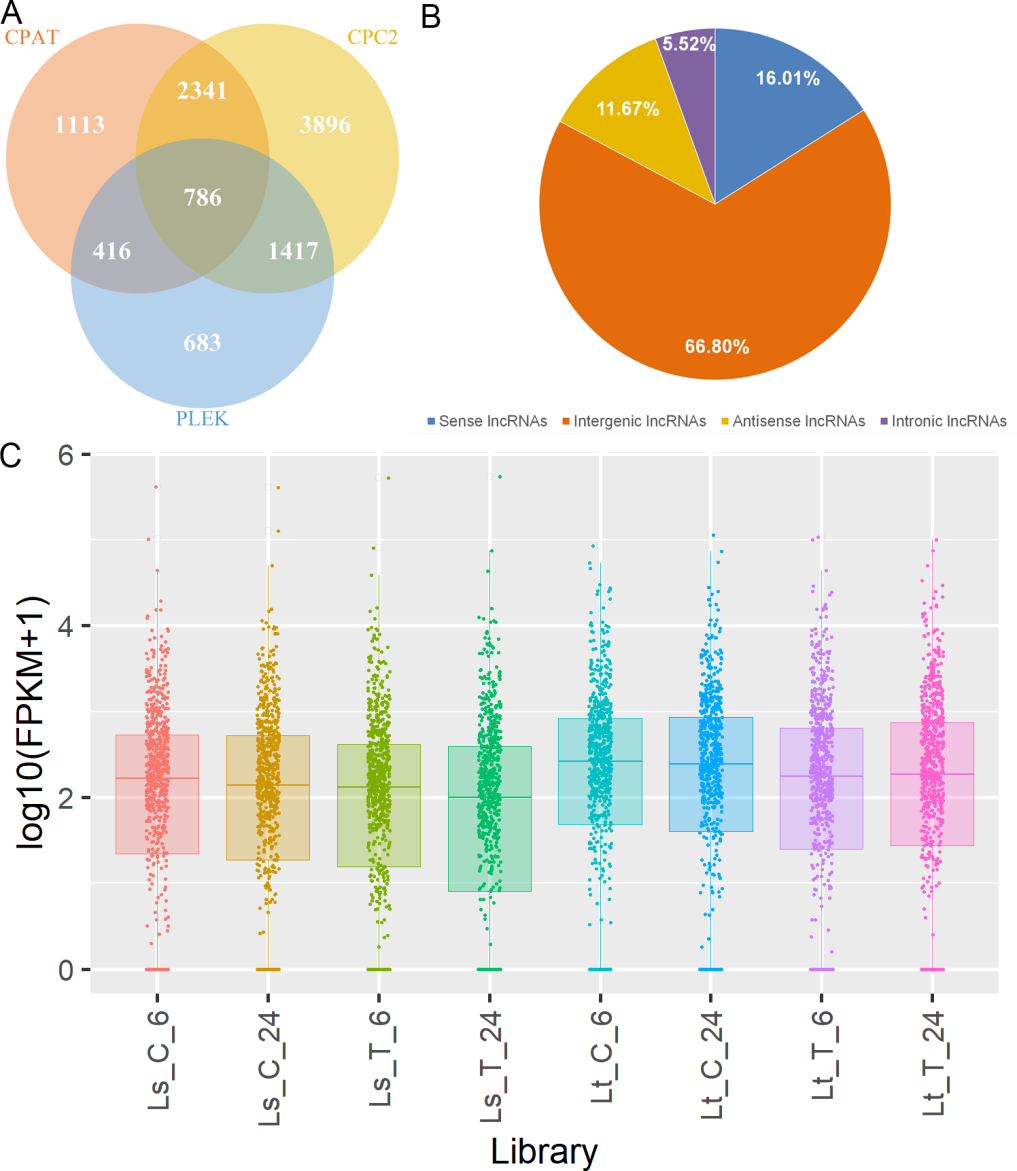
Summary of the lncRNA identification and quantification. **(A)** Potential lncRNAs identified through the three different approaches: CPC2 (yellow), CPAT (orange), and Plek (blue); and the identified lncRNAs common for different pairs and all three approaches. **(B)** Classification of identified lncRNAs into four classes: intergenic (orange), intronic (purple), sense (blue), and antisense lncRNAs (yellow). **(C)** lncRNA FPKM distribution across the libraries (Ls_C_6, Ls_C_24, Ls_T_6, Ls_T_24, Lt_C_6, Lt_C_24, Lt_T_6, Lt_T_24).

After the transcripts were assembled, they were filtered and only the ones longer than 200 nt, with the expression level higher than 0.1, containing more than one exon and not overlapping with known protein-coding genes were kept, leaving only the 24,677 transcripts with class codes “u”, “i”, “o” and “x”. Three different approaches were then applied to identify lncRNAs among those transcripts: CPC2, CPAT, and Plek. Only the 786 transcripts determined to lack any coding potential by all three methods were considered as reliably expressed lncRNAs (**Figure 5A**, **Supplementary Table S6**). Comparisons were made between the lncRNA and coding gene positions in the genome showing that 66.8% were intergenic lncRNAs (lincRNAs) (**Figure 5B**). Also, comparing lncRNA to coding genes revealed that lncRNA genes had fewer exons than the coding ones. Single-exon lncRNA genes comprised 40.1% of all lncRNA genes, while those with two exons took up 55.2%. On the other hand, coding genes with one and/or two exons made up only 37.2%, while genes with three or more exons took up 62.8%. Additionally, 62.9% of lncRNAs were <500 nt, and mRNAs were of similar length – 54.4% were <500 nt. However, the percentage of those longer than 1000 nt was higher in the mRNA group (17.5%), compared to lncRNAs (4.2%).

**Figure 6:**
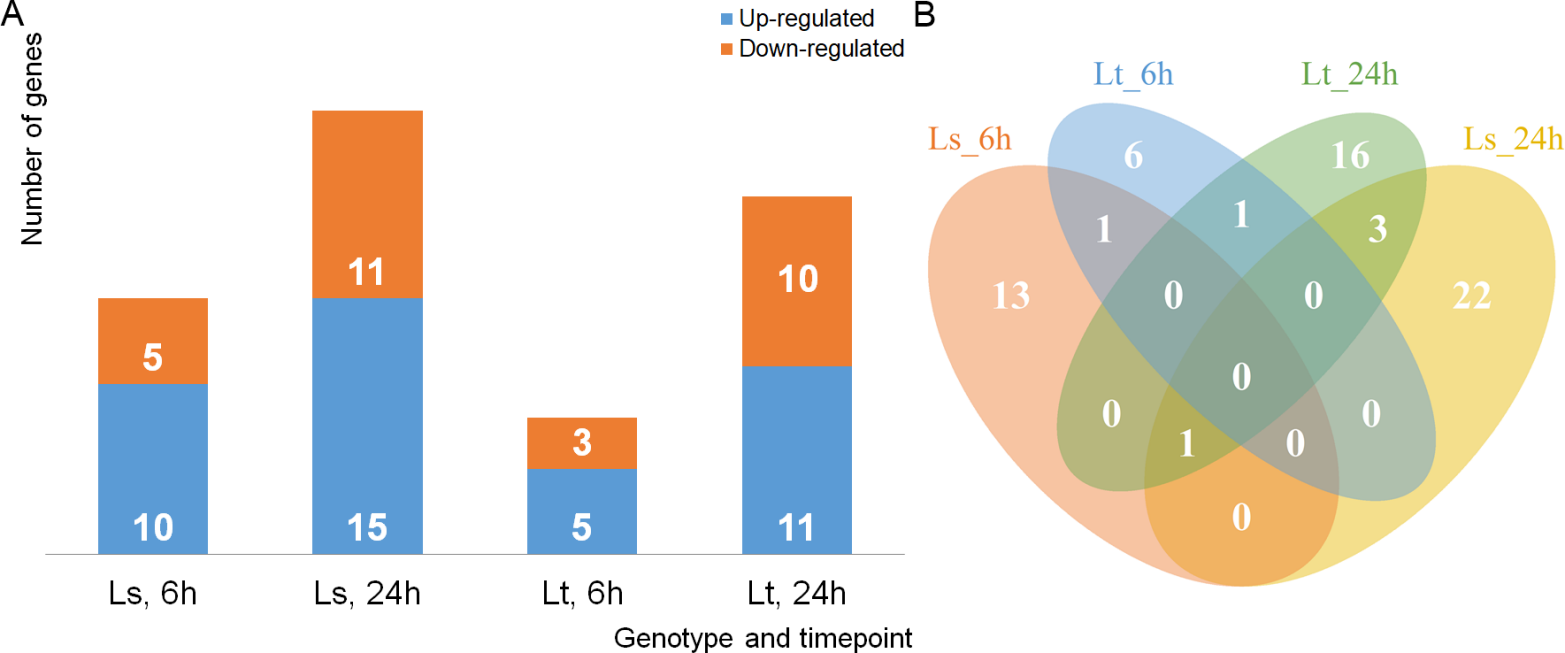
Differentially expressed lncRNA. **(A)** lncRNAs differentially expressed between the control and treatment, in the two genotypes (L_S_, L_T_), at the two time points (6h and 24h). Up-regulated lncRNAs are shown in blue, while down-regulated are in orange. **(B)** Unique and common differentially expressed lncRNAs between the two genotypes (L_S_, L_T_) and time points (6h and 24h). Ls_6h is shown in orange, Ls_24h in yellow, while Lt_6h is presented in blue, and Lt_24h in green.

Quantification of lncRNAs was performed in htseq and both raw counts and FPKM values were calculated (**Figure 5C**). DE lncRNAs under LT stress were calculated through the edgeR package and 63 lncRNAs, between the control and treatment samples, were identified across the two genotypes and time points (**Figure 6A**, **Supplementary Table S7**). Unlike the mRNAs, no lncRNA was common for both genotype and treatment duration (**Figure 6B**). More than half were found to be DE in only one of the time points in a single genotype. XLOC_000175 was the only lncRNA DE in both genotypes after 6h: L_T_-6h logFC = 2.13; L_S_-6h logFC = 2.28. On the other hand four DE lncRNAs were common for both genotypes after 24h: XLOC_001043 (L_T_ logFC = 2.38, L_S_ logFC = 3.83), XLOC_006714 (L_T_ logFC = 11.65, L_S_ logFC = 4.67), XLOC_015129 (L_T_ logFC = 2.88, L_S_ logFC = 3.08), and XLOC_016664 (L_T_ logFC = 6.20, L_S_ logFC = 3.69). WGCNA analysis showed a single cluster of 153 lncRNAs associated with the LT treatment, in which 41 lncRNAs showed high intramodular connectivity (p < 0.05, **Supplementary Table S8**). Among the DE lncRNAs, eight were found to be potential hub genes in the module associated with the LT treatment (XLOC_000816, XLOC_001043, XLOC_008440, XLOC_009662, XLOC_010836, XLOC_011976, XLOC_012259, XLOC_016214).

Gene targets of lncRNA were identified using two methods: potential *cis*-targets were searched for 100 kb up- or downstream using bedtools, and the *trans*-targets were predicted with the LncTar software. A total of 362 potential *cis*-targets of 63 DE lncRNA were discovered (**Supplementary Table S9**), with targets of only five lncRNAs being DE: XLOC_009238-Zm00001eb069040, XLOC_016169-Zm00001eb164390, XLOC_010836-Zm00001eb113210, XLOC_006594-Zm00001eb420380, XLOC_015072-Zm00001eb180830. Additionally, only three had Uniprot characterizations: *rca* (Zm00001eb164390), *tic32* (Zm00001eb420380), and *sweet* (Zm00001eb180830). XLOC_016169 appeared to have a negative effect on the expression of *rca* in L_T_, after 24h of LT exposure; while XLOC_006594 and XLOC_015072 showed a positive correlation with their target genes, *tic32* and *sweet*, respectively – both pairs were down-regulated after 24h. On the other hand, 191 potential *trans*-targets of 22 DE lncRNAs were discovered (**Supplementary Table S10**). Only 66 of the potential target mRNAs were DE. Most significantly, the target analysis showed that XLOC_012388 positively influenced the expression of *hsp17*. Also, *elip2* was shown to be targeted by three different lncRNAs (XLOC_016783, XLOC_002167, and XLOC_006091) in the two genotypes. These lncRNAs were upregulated after 6h, and seem to positively influence *elip2* expression as well. psRobot was used to identify potential lncRNA targets of previously identified miRNAs expressed in LT conditions of 5-d old seedlings (Božić et al., 2024), and it has been identified that nine miRNAs may potentially have an effect on seven lncRNAs (**Supplementary Table S11**). Only XLOC_009553, targeted by zma-miR166k-5p, was DE. However, zma-miR166k-5p was not shown to be DE in Božić et al. (2024).

### 3.4 Identification and characterization of LT-responsive circRNAs in maize

**Figure 7:**
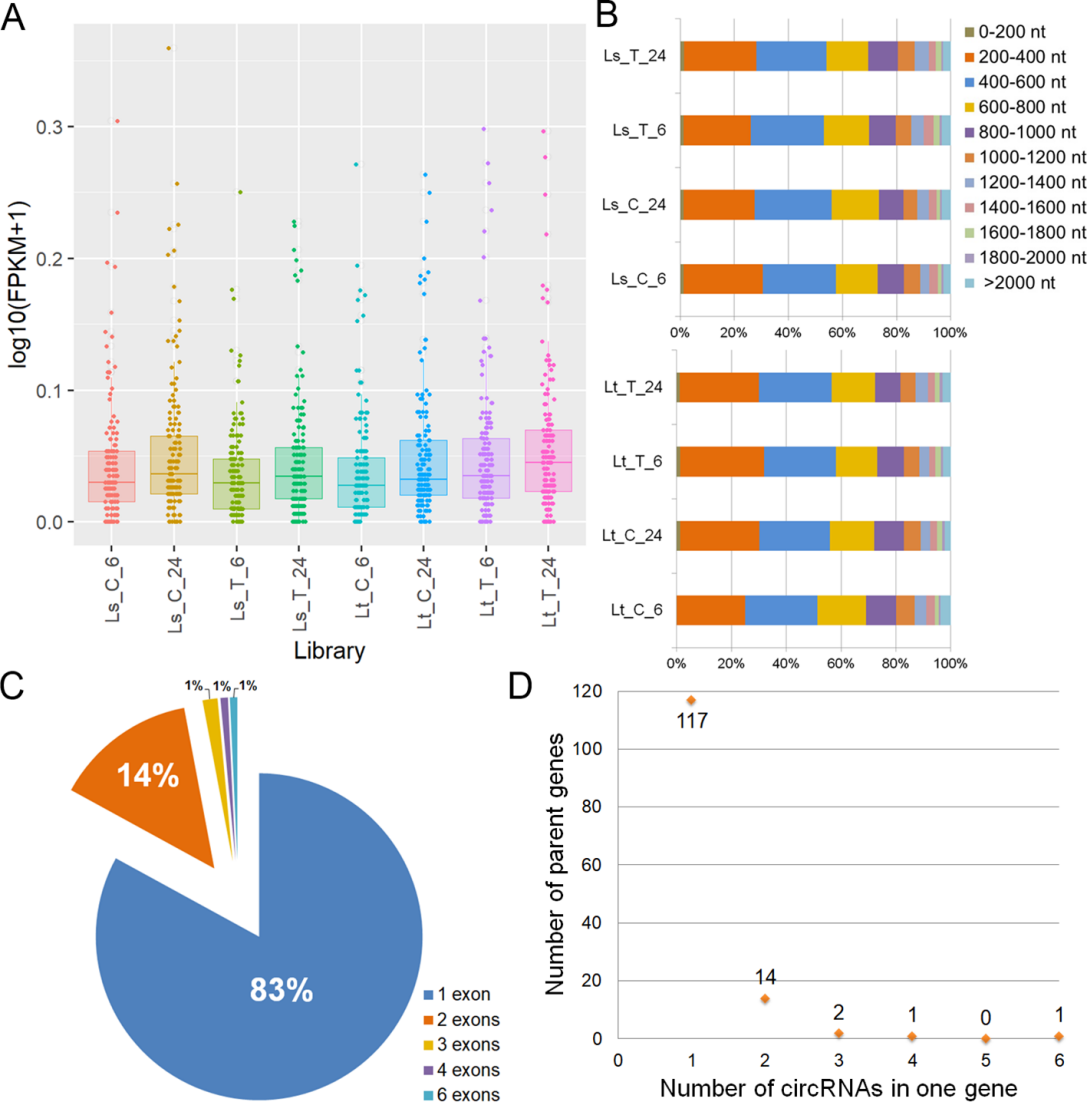
Identified circRNAs. **(A)** circRNA FPKM distribution across the samples (Ls_C_6, Ls_C_24, Ls_T_6, Ls_T_24, Lt_C_6, Lt_C_24, Lt_T_6, Lt_T_24) of the selected 135 circRNAs. **(B)** Length distribution across the eight libraries of the selected 135 circRNAs. **(C)** Exon number of the selected 135 circRNAs. 83% of circRNAs had one exon and are shown in blue, those with two accounted for 14% and are shown in orange, while the rest of circRNAs were represented with 1% in each category: circRNAs with three exons shown in yellow, those with four in purple and six in light blue. **(D)** The number of parent genes (y-axis) that generated different numbers of circRNAs (x-axis).

The circRNAs involved in the LT response in 5d-old seedlings were identified, along with the alternative back-splicing sites using CIRCexplorer2. Additionally, the circRNAs were quantified through htseq and only the ones with FPKM > 0.1 in at least one library were considered for further analysis. These included 135 identified circRNAs (**Figure 7A**, **Supplementary Table S12**). Most of the identified circRNAs were 200-800 nt in length (**Figure 7B**). Exonic circRNAs made up the most of expressed circular RNAs (46.94%), while the intronic and intergenic comprised 32.43% and 20.63%, respectively. Nearly 83% were single-exon circRNAs, while the rest contained up to six internal exons (**Figure 7C**). Also, 86.67% of circRNAs were produced by a single parent gene. Two circRNAs from one gene accounted for 10.37%, and three different circRNAs made up 1.48%. Only two genes were responsible for forming more than three circRNA isoforms: Zm00001eb224050 generated four circRNAs, while Zm00001eb420520 held six different circRNAs (**Figure 7D**). edgeR was used to detect DE circRNAs between the treated and control samples, but none were found. Additionally, 11 microRNAs were found to potentially target 11 circRNAs (**Supplementary Table S13**).

### 3.5 Construction of the lncRNA/circRNA-miRNA-mRNA network

Non-coding RNAs have the ability to target coding RNA molecules and regulate their expression through different mechanisms. The lncRNA/circRNA-miRNA-mRNA networks can be constructed based on these interactions, so first each of the individual interactions between RNA classes and their correlations had to be predicted. Potential lncRNA-miRNA, lncRNA-mRNA, and circRNA-miRNA interactions were already described in previous sections. Potential mRNA-miRNA interactions were also analyzed in Božić et al., 2024.

**Figure 8:**
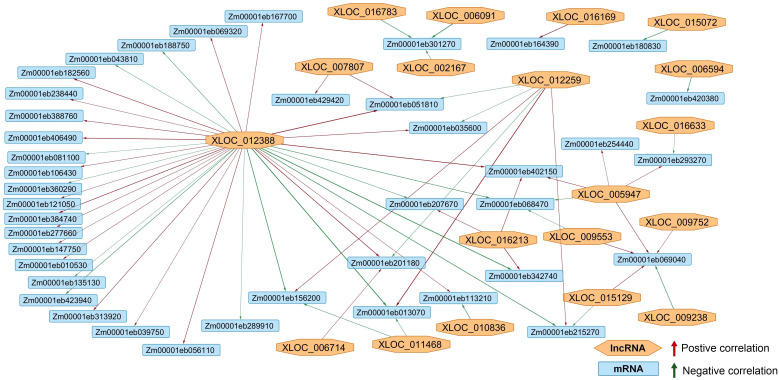
The lncRNA-mRNA coexpression network. Orange nodes represent the lncRNAs, while the blue rectangles represent the target mRNAs. Positive correlation between the lncRNA and mRNA expression is shown with a green arrow, while the negative is shown with a red one. The network showed that the expression of 19 lncRNAs was correlated to the expression patterns of 41 target mRNA.

Due to the lack of differentially expressed target RNAs (circRNAs and lncRNA-targets of miRNAs), the only parts of the network that could be predicted were lncRNA-mRNA and miRNA-mRNA. The network showed that the expression of 19 lncRNAs was correlated to the expression patterns of 41 target mRNA (**Figure 8**). XLOC_012388 acted as a hub-regulator, affecting the largest number of target genes. Most of the XLOC_012388 target genes had no functional Uniprot annotation, but the ones that did were involved in growth and development (protodermal factor 1; zinc finger protein 2; small auxin up-regulated protein 37, SAUR37), photosynthesis (oxygen-evolving enhancer protein 3-1, sedoheptulose-1,7-bisphosphatase), and abiotic stress response (hsp17, hsp26). The *elip2* gene (Zm00001eb301270) was possibly positively regulated by three different lncRNAs: XLOC_016783, XLOC_002167 and XLOC_006091. Giberellin-regulated protein 1 gene’s (Zm00001eb051810) expression was also modified by three lncRNAs: XLOC_012259, XLOC_007807, XLOC_012388. Still, most of the genes targeted by more than one lncRNA, such as Zm00001eb069040, Zm00001eb215270, Zm00001eb293270, Zm00001eb156200, did not have functional annotations.

A total of 1822 genes were identified as targets for both known and novel miRNAs. Seven miRNAs and the target mRNAs were found to have opposite expression patterns, pointing to the possible role of the miRNAs in gene expression regulation (**Supplementary Table S14**). Two potentially novel miRNAs seemed to target genes important for the Calvin cycle: down-regulation of novel_452 had a positive effect on the *rbcx2* expression, while novel_696 was up-regulated leading to decreased expression levels of *prk*. On the other hand, some known miRNAs seem to be directly involved in the LT response. zma-miR164a-3p was upregulated after 24h in L_S_, and the predicted target gene, *HSFBa*, was significantly down-regulated.

### 3.6 qRT-PCR validation of the sequencing results

Sequencing results were validated through qRT-PCR analysis of the selected DE mRNAs and lncRNAs. Six mRNAs (*ADO3, THI1, AED1, nat4, pds, rca*) and five lncRNAs (XLOC_012565, XLOC_000175, XLOC_001043, XLOC_006714, XLOC_016783) were selected for the analysis. In general, the expression patterns obtained through qRT-PCR matched those obtained through NGS sequencing, with small inconsistencies (for example, in L_T_-6h, changes in expression of *aed1* and *thi1* were detected in qRT-PCR, but not in sequencing results). Results of the qRT-PCR validation of mRNAs are shown in **Supplementary Figure S1**, while lncRNA validation results are shown in **Supplementary Figure S2**.

## 4 Discussion

### 4.1 Low temperatures affect various photosynthesis parameters in 5-day-old maize seedlings

Photosynthesis is one of the most important metabolic processes in plants, and is crucial for successful growth and development, as well as the plant yield (Muhammad et al., 2021). However, it is significantly affected by various abiotic stressors, including low temperatures (Gururani et al., 2015). This is particularly true for C-4 plants, such as maize, that have a more sensitive photosynthetic apparatus (Bilska-Kos et al., 2018). Low temperatures affect photochemical efficiency of the PSI and PSII, chlorophyll biosynthesis, Calvin cycle enzyme activity, particularly Rubisco (Muhammad et al., 2021). Suboptimal temperatures also lead to the generation of reactive oxygen species (ROS) (Nishiyama and Murata, 2014), which cause further oxidative damage to the photosystems and inhibition of PSII repair (Nishiyama et al., 2011; Gururani et al., 2015). This research found, that by affecting the expression of certain genes, the LT treatment in 5-day-old seedlings had an effect on the PSI and PSII assembly, light-harvesting complex (LHC) stability, and Rubisco activity. Additionally, the treatment temperatures activated certain defensive responses when it comes to oxidative damage caused by the destabilization of the photosynthetic apparatus.

#### 4.1.1 LT impact on both photosystems

LT affected the integrity of both photosystems in the 5d-old maize seedlings. Genes encoding structural proteins of both PSI and PSII reaction centers, and the components of the antenna complexes were down-regulated in both genotypes. The down-regulated genes included: *psa* genes necessary for PSI reaction center assembly (Ozakca, 2013); *psb* genes required for PSII assembly and stability (Pagliano et al., 2013); and several genes responsible for the expression of chloroplastic chlorophyll a-b binding (CAB) proteins of the LHC. CAB proteins comprise the antenna LHC of both PSI and PSII (Zhao et al., 2020). LHC are necessary for the initiation of photochemical reactions, maintaining thylakoid membrane structure, and regulating excitation energy distribution between PSII and PSI (Gan et al., 2019). Down-regulation of the *psa, psb, lhc*, and *cab* genes in LT conditions has been reported before in various plant species, including maize (Velitchkova et al., 2020; Yu et al., 2021; Banović Đeri et al., 2022; Balassa et al., 2022), implying that the light-dependent reactions are particularly sensitive to LT.

#### 4.1.2 Calvin cycle enzymes affected by LT

Several genes involved in the regulation of the Calvin cycle and Rubisco activity were found to be DE. Rubisco activity is a key factor in determining the rate and proper functioning of photosynthesis under both optimal and LT conditions, particularly in C4 plants. C4 plants have lower levels and capacities of Rubisco compared to C3 plants (Salesse-Smith et al., 2020). Therefore, maintaining adequate Rubisco activity is essential for sustaining the photosynthesis rates needed for survival under environmental stress. Rubisco activase (RCA), encoded by the *rca* gene, is the enzyme responsible for regulating Rubisco (Bracher et al., 2017). Up-regulation of *rca* leads to higher photosynthesis rates and productivity (Zhang et al., 2019c; Feng et al., 2023). Data on the effect of low temperatures on *rca* expression is lacking, but under other abiotic stressors, *rca* expression was increased in maize (Salesse-Smith et al., 2018; Sparrow-Muñoz et al., 2023). Here, in LT conditions, *rca* was down-regulated after 24h in both genotypes. Interestingly, *rbcx2 gene*, encoding one of the proteins necessary for the Rubisco assembly, chloroplastic chaperonin-like RbcX protein 2 was upregulated after 6h of treatment in both genotypes. The RbcX proteins are known to have chaperone activity during the assembly of Rubisco in cyanobacteria (Li et al., 2022), but their homologues were found in higher plants as well, where they fulfill a similar role (Kolesiński et al., 2011). To the authors’ knowledge this is the first note of any *rbcx* gene being DE under abiotic stress conditions. Additionally, phosphoribulokinase gene, *prk*, was down-regulated after 24h. PRKs are a part of the regeneration phase of the Calvin cycle (Gurrieri et al., 2021). Again, the data on *prk* expression levels under abiotic stress in maize is lacking, but the gene’s down-regulation was reported in cold-treated Iranian wheat (Rinalducci et al., 2011). *prk* was also down-regulated in salt and drought-treated wheat (reviewed in Kosová et al., 2015).

The results show that LT negatively affected the light-independent phase of photosynthesis in 5-day-old maize seedlings, by limiting carbon fixation and ribulose regeneration in the Calvin cycle, regardless of genotype. However, in the first 6h of treatment, a course of action was taken to alleviate the described negative effects by intensifying the assembly of Rubisco, through *rbcx2* up-regulation. The expression levels of *rbcx2* were significantly higher in L_T_, offering a possible explanation for a better recovery of the Calvin cycle after 24h in the tolerant genotype.

#### 4.1.3 LT-induced photoinhibition and defensive mechanisms

The damages to both photosystems and the reduction of the Calvin cycle enzyme activity, can lead to notable decreases in photosynthetic efficiency. Lowered photosynthetic efficiency results in inhibited electron transport and an increase in ROS levels and photoinhibition (Gan et al., 2019). This further leads to the over-excitation and loss of structural integrity of PSII (Tikkanen and Aro, 2014), as well as to the hindrance of PSII damage repair (Nishiyama and Murata, 2014). Protein responsible for the PSII repair, protein D1, is encoded by the *psbA* gene (Gururani et al., 2015). Interestingly, while the *psbA* gene was not found to be DE, a gene encoding the carboxyl-terminal-processing peptidase 2 (*CTPA2*), necessary for the C-terminal processing and activation of the D1 protein (Che et al., 2013) was upregulated in both genotypes after 6h. This finding suggests that, while the synthesis of protein D1 did not differ between the control and treatment conditions, it was activated only under low-temperature conditions as a way of ensuring damage repair of PSII.

Besides the PSII repair, additional protective measures were likely employed to alleviate the harmful effects associated with ROS and photoinhibition. For example, early light-inducible proteins (ELIPs) protect chloroplasts from photodamage, by facilitating energy dissipation that protects PSII from photoinhibition (Liu et al., 2020). Low temperatures were reported to lead to ELIP up-regulation in Arabidopsis (Hayami et al., 2015), and *Medicago sativa* (Zhuo et al., 2013), but no data exists for maize. Herein, *elip2* was up-regulated in L_S_ and both *elip1* and *elip2* in L_T_ after 6h of treatment. Rizza et al. (2011) showed that in Arabidopsis, *elip1* and *elip2* were independently regulated under abiotic stress conditions. This coincides with the results in 5d-old seedlings, since *elip1* was upregulated only in L_T_ and suggests a factor possibly involved in establishing low temperature tolerance in maize seedlings. Additionally, two glutaredoxin genes (*GRX*) were upregulated after 6h: *GRXS17* in L_S_, and *GRXS5* and *GRXS17* in L_T_. Glutaredoxins (GRXs) are oxidoreductases that are involved in controlling the redox potential and ROS regulation (Meyer et al., 2009). *GRXS17* genes were found to be upregulated in Arabidopsis during heat stress (Martins et al., 2020; Rao et al., 2023), and *AtGRXS17* overexpression in tomato lead to enhanced chilling tolerance (Hu et al., 2015). *GRXS5* was also involved in abiotic stress response in the fern species *Pteris vittata* (Sundaram et al., 2008), and overexpression of *PvGRXS5* in Arabidopsis increased the plants’ tolerance to heat stress and reduced oxidative damage to proteins (Sundaram and Rathinasabapathi, 2010). It appears that GRXs fulfill a similar role in 5d-old maize seedlings, suggesting a possible role in modulating responses to extreme temperatures in different crops. The difference in their expression levels between L_S_ and L_T_ again alludes to the possible role of antioxidative responses being important for establishing chilling tolerance. To the author’s knowledge, this is the first report on their differential expression in maize under low-temperature conditions.

Another activated photoprotective measure was the antioxidative pigment accumulation, including carotenoids and anthocyanins. Carotenoid levels are increased during abiotic stress periods as a way to limit the damages of ROS (Uarrota et al., 2018). Various plant species displayed increased carotenoid levels under low temperatures (Guo et al., 2022; He et al., 2023), including maize (Obeidat et al., 2018; Waititu et al., 2021). Several enzymes involved in the carotenoid biosynthetic pathway were up-regulated in both genotypes of 5-d old seedlings. The *psy2* gene is one of the three phytoene synthases found in maize, and it was upregulated in both genotypes after 6h. The PSY2 enzyme is responsible for carotenoid accumulation in green tissues where the photosynthetic processes occur (Zhou et al., 2022b). The *pds* gene encoding the 15-cis-phytoene desaturase, that yields 9,15,9’-tri-cis-ζ-carotene (Koschmieder et al., 2017), was up-regulated in both genotypes after 24h. An interesting difference between the genotypes was the apparent increase in anthocyanin content only in L_T_, through the up-regulation of an enzyme involved in the biosynthetic pathway: anthocyanidin 3-O-glucosyltransferase (UF3GT). *UF3GT* expression was increased in the treatment conditions at both time points in L_T_. Anthocyanin accumulation was connected to enhanced protection from photoinhibition in maize leaves at low temperatures (Rodríguez et al., 2014; Yu et al., 2022).

In conclusion, the findings of changes in expression regarding photosynthesis-related genes, show that LT significantly affected the photosynthetic process regardless of genotype and their susceptibility to this stress factor: by affecting the PS assembly and their photorecepting units (LHC), as well as the activity of crucial enzymes, like Rubisco. However, the difference in low-temperature susceptibility between the genotypes in the VE stage could be attributed to the courses of action taken to limit the damage caused by LT-induced ROS accumulation and photoinhibition. L_T_ seemed to be more efficient in handling the oxidative damages: with higher expression levels of proteins and enzymes with antioxidative roles, such as *CTPA2, ELIPs, GRXs, UF3GT* (**Supplementary Figure S3**).

### 4.2 Genes involved in the temperature stimuli response differed among the two genotypes after the LT treatment

GO analysis showed that the DE genes enriched in the response to abiotic stimuli, encompassed reactions to both heat and cold. Most of the genes belonged to the HSP and HSF family. Interestingly, nearly every identified *hsp* or *HSF* gene was down-regulated. The only exception was *hsp17*, up-regulated in L_S_ after 24h. Still, a larger number of down-regulated *hsp/HSF* genes were identified in L_S_, rather than L_T_. LT is most often associated with the accumulation of HSPs, as a way to decrease protein dysfunction and denaturing (Hlaváčková et al., 2013; ul Haq et al., 2019), but this is not the case for every plant species. There are reports of both HSPs and HSFs up-regulation (Kollipara et al., 2002; Meng et al., 2022), as well as their down-regulation in maize (Li et al., 2020; Sowiński et al., 2020). Sowiński et al. (2020) compared several independent transcriptomic research studies focusing on the response of maize seedlings to moderate (12–15°C) or severe cold stress (below 8°C). They found that in both cases *hsp* genes were mostly down-regulated, but the number of DE *hsp* genes was higher in maize plants subjected to severe cold conditions. Unlike L_T_, expression patterns in L_S_ were more similar to those found in studies with maize subjected to temperatures lower than 8°C, suggesting the temperatures applied in this research affected the susceptible genotype more gravely. Since the up-regulation of HSPs is often considered a crucial aspect of the plant’s response to any abiotic stressor (Janmohammadi et al., 2015), it would be safe to assume that the inability to limit protein denaturation and dysfunction, can be a significant factor in maize susceptibility to low temperatures, as well as in the differences in susceptibility between the different maize genotypes.

Two DE genes were enriched for response to cold: thiamine thiazole synthase, *THI1*, and the previously mentioned, *pds*, involved in carotenoid biosynthesis. THI1 is involved in the biosynthesis of thiazole, a precursor of thiamine (vitamin B_1_). Thiamine is involved in various metabolic processes such as glycolysis, pentose phosphate pathway, and the tricarboxylic acid cycle (Yusof, 2019), but also abiotic stress response (Yee et al., 2016). *THI1* levels were shown to be increased under drought response in Arabidopsis (Li et al., 2016), and *Medicago sativa* (Yin et al., 2022). The down-regulation of *THI1* only in L_S_ can also be a factor in the difference in LT susceptibility between genotypes. To the author’s knowledge, this is the first description of *THI1* involvement in LT response in maize.

### 4.3 lncRNAs appeared to affect both growth and development, in addition to the low-temperature response

lncRNAs are known to have important roles in the growth and development of plants, as well as responses to environmental changes, including low temperatures (Biswas et al., 2021). Many lncRNAs have been identified under LT stress in numerous plant species: Arabidopsis (Calixto et al., 2019), rice (Leng et al., 2020), wheat (Lu et al., 2020). lncRNAs expressed under abiotic stresses have been researched in maize as well: under conditions of drought (Pang et al., 2019), heat (Hu et al., 2022), nitrogen deficiency (Ma et al., 2021b). The involvement of lncRNAs in the low temperature response has also been studied in V_6_ maize seedlings (Waititu et al., 2021) and primary root tips of 8-day-old seedlings (Xuhui et al., 2022). To the author’s knowledge, this is the first study of lncRNAs in 5-day-old maize seedlings.

Herein, 786 transcripts were determined to satisfy the necessary criteria to be considered lncRNAs. A large number of the transcripts were designated as lincRNAs, each less than 1000 nucleotides in length, which corresponds with the findings of Xuhui et al. (2022). Additionally, lncRNA genes with 1-2 exons comprised more than 95% of all expressed lncRNAs, a much higher ratio than in mRNA genes (37%). Similar results were reported in other works related to maize lncRNAs under abiotic stress (Yu et al., 2020; Hu et al., 2022; Liu et al., 2022b). Of the 63 DE lncRNAs, more than half were found to be DE in only one of the time points in a single genotype and none were common for both genotypes and time-points. Also, the lncRNA expression levels were significantly lower than the levels of expression of mRNAs, as reported in previous studies. Further characterization of identified and DE lncRNAs was made difficult due to the lack of specialized databases for plant lncRNAs. For example, the most widely used database PLncDB (Jin et al., 2021), contains more than 30,000 entries for maize but only 26 are validated.

lncRNA research is still in the phase of large-scale identification, rather than functional characterization (Gonzales et al., 2024). Still, some of their functions, particularly through their interaction with other RNA classes, such as miRNAs, are known. Since none of the miRNA-lncRNA pairs were DE, making any conclusions about the interaction is difficult. lncRNAs can also directly target the expression of genes positioned in the vicinity of their transcription sites in a *cis*-manner, regulating genes found at or near the same genomic locus, or in a *trans*-manner, at independent chromosomal loci (Fatica and Bozzoni, 2014; Waseem et al., 2021). Regarding *cis*-targets, XLOC_016169 negatively impacted *rca* expression in L_T_, suggesting its role in regulating Rubisco activity. XLOC_006594 seemed to positively influence *tic32* (chloroplastic protein TIC32) expression; while XLOC_015072 also showed positive expression correlation with target gene *sweet* (bidirectional sugar transporter, SWEET). TIC32 is a short-chain dehydrogenase, part of the TIC (translocon of the inner chloroplast envelopes) complex, essential for chloroplast biogenesis (Hörmann et al., 2004). There is no data on *tic32* expression under low-temperature conditions, but under heat stress, it was reduced in pea (Dutta et al., 2009). SWEET has been shown to have an important role in low-temperature tolerance in several plant species (Miao et al., 2017; Zhang et al., 2019a). Their down-regulation in L_S_ only may point to their role in establishing low temperature tolerance in maize lines.

Potential DE *trans*-mRNA targets were involved in growth and development (germination, tissue differentiation; Casparian strip development), abiotic stress response (heat shock proteins, antioxidative response), and protein degradation. This result suggests the possibility of maize lncRNAs being directly involved in the plants’ response to LT, by affecting the expression of genes involved in the stress response (*rca, elip2, hsp17*), but confirmation of this assumption would require further characterization of the lncRNAs and exploration of their role in this pathway.

### 4.4 circRNAs were not differentially expressed under LT conditions in maize at the analyzed stage

circRNAs have been identified in various plant species, such as Arabidopsis (Ye et al., 2015), rice (Zhou et al., 2021), wheat (Han et al., 2021), and maize (Han et al., 2020; Xu et al., 2024). circRNAs have been shown to be involved in the response of maize plants to drought (Zhang et al., 2019b; Xu et al., 2024), increased salt levels (Liu et al., 2022b), soil nitrogen deficiency (Ma et al., 2021a). The involvement of circRNAs in the maize cold response was described only in a meta-analysis done by Tang et al. (2018). To the authors’ knowledge this is the first description of their identification and expression in 5-day-old maize seedlings.

Initial identification revealed 6951 circRNAs. However, nearly 91% had expression levels lower than the set threshold, meaning they were present with less than 10 reads across all libraries. To ensure reliable expression analysis, such transcripts were not considered as expressed circRNAs with high confidence and were not regarded in downstream analyses, leaving 135 circRNAs for further analyses. Most of the identified circRNAs were 200-800 nt in length, which is in line with other research works regarding maize circular RNAs, as well as those of other plant species (Han et al., 2020; Ma et al., 2021a). On the other hand, the largest ratio of the expressed circRNAs was exonic circular RNAs. In maize, the classification of circRNAs based on the positional relationship between the circRNAs and their parent gene is not uniform. There are reports of different ratios of exonic, intronic, and intergenic circRNAs, but in all circRNAs are mostly of exonic origin, with intronic and intergenic circRNAs varying in proportion (Ma et al., 2021a; Xu et al., 2024). Alternative back-splicing is a process in which multiple circRNAs are derived from the same back-splice site in a single gene (Tang et al., 2018). Despite detecting 63 unique back-splice junctions in the 5d-old maize seedlings, nearly 90% of circRNAs were produced from a single gene. Parent genes of multiple circRNAs were responsible for the formation of less than 15% of all circRNAs. Again, there are multiple reports of alternative back-splicing events in maize, with the percentage of single-gene-origin circRNAs covering different proportions of total circRNAs. However, findings similar to the ones from this research were reported by Luo et al. (2019) and Liu et al. (2022b). When it comes to the involvement of identified circRNAs in the LT response, no DE circRNAs were detected in the 5d-old maize seedlings. Also, despite identifying eleven miRNAs potentially targeting identified circRNAs, the target circRNAs, nor their parent genes, were DE. For this reason, no conclusions could be drawn about the role of miRNAs in regulating circRNAs under chilling conditions.

Circular RNAs are known to be expressed at levels lower than linear RNAs in plants and animals (Wang et al., 2014; Liu et al., 2023). Still, expression levels detected in this research are even lower than those reported in existing literature. For example, Han et al. (2020) showed that in V3 and V5/V6 maize seedlings ≈70% of circRNAs were expressed with less than ten reads across all libraries, which is lower than the ratio identified in this experiment (91%). A possible explanation for this finding could be the examined vegetative stage: five days post-germination did not seem to be enough time for circRNAs to accumulate and be effectively included in the low-temperature response.

### 4.5 Lack of DE ncRNAs complicated the lncRNA/circRNA-miRNA-mRNA network construction

Potential interactions between the lncRNAs and other RNA classes (miRNA, mRNA) needed for the lncRNA/circRNA-miRNA-mRNA network construction, were already described in previous chapters, just like circRNA-miRNA associations.

zma-miR164a-3p negatively impacted the *HSFBa* expression. miR164a-3p were previously identified to participate in the salt treatment response in maize (Fu et al., 2017) and tomato (Wang et al., 2021). However, miR164a-3p were down-regulated in both plant species under the salt treatment. To the authors’ knowledge, this is the first time their role in targeting heat stress factors under the chilling treatment was described in maize. Despite finding significant miRNA-mRNA interactions, the target pairs did not form a network, but were all separate entities: there were no individual miRNAs found that affect multiple genes, nor target genes regulated by several miRNAs.

The lncRNA-mRNA coexpression network comprised of 19 lncRNAs and 41 target mRNAs (**Figure 8**). As previously stated, lncRNAs can affect target genes through multiple mechanisms, including directly binding to mRNAs to regulate the mRNA stability, interfering with transcription, as well as acting as miRNA sponges as explained in the ceRNA hypothesis (He et al., 2020), resulting in different co-expression patterns, seen here as well. lncRNA XLOC_012388 acted as a hub-regulator, affecting the largest number of target genes. lncRNAs affected the expression of genes involved in growth and development, photosynthesis, and abiotic stress response. Still, most of the genes targeted by the lncRNAs, did not have functional annotations, which made drawing any conclusions about the proposed regulatory network more difficult.

Due to the lack of DE circRNAs and the lncRNA-targets of miRNAs not being DE, constructing the complete lncRNA/circRNA-miRNA-mRNA network was difficult, despite many potential cases. For example, the target prediction showed that several lncRNAs (XLOC_005314, XLOC_010450) and circRNAs (bna_circ_824, bna_circ_1505) have binding sites for zma-miR169i-3p, which they share with two target genes (Zm00001eb339510, Zm00001eb286120). This suggests a possible role of those non-coding RNAs in modulating the expression of target mRNAs, but it could not be confirmed due to the lack of expression data. A possible explanation could be the analyzed developmental stage in which lower expression levels, particularly of the noncoding RNA molecules, were detected.

## 5 Conclusion

The impact of low temperatures on the levels of coding and long non-coding RNAs in 5-day-old maize seedlings, during the VE stage, is substantial, allowing for conclusions to be drawn regarding the plants’ responses and the distinctions between tolerant and susceptible genotypes. According to the results, photosynthesis is the process most affected by low temperatures. The LT treatment had an effect on the integrity of the reaction centers and antenna complexes of both photosystems, as well as on the Calvin cycle enzyme activity and chloroplast assembly, by affecting the expression of various important genes (various *psa, psb, lhc, cab* genes; *rca*; *prk; tic32*). Since such a decrease in photosynthetic efficiency can lead to the generation of ROS and photoinhibition, it was not surprising that many genes involved in limiting the photooxidative damage were upregulated (*ctpa2, grxS17, grxS5, elip1, elip2, UF3GT*). It was in the expression of these genes where differences between the genotypes could be seen: according to the results, L_T_ seemed better equipped at fighting photoinhibition through a more significant up-regulation of these genes. Additionally, the expression of many of the genes seemed to be regulated by non-coding RNA: XLOC_016169-*rca*; XLOC_006594-*tic32*; XLOC_016783-XLOC_002167-XLOC_006091-*elip2*; novel_452-*rbcx2*; novel_696-*prk*. However, the low levels of non-coding RNA expression in this developmental stage prevented the complete lncRNA/circRNA-miRNA-mRNA network from being formed. Certain genes’ roles in the low-temperature or abiotic stress response in maize weren’t previously described: *rbcx*, *grxS17, grxS5, THI1*; and neither were the interactions with lncRNAs.

This research is the first of its kind to be carried out during such an early developmental stage, such as VE, under LT conditions in maize. Additionally, to the authors’ knowledge it is the first study of lncRNAs and circRNAs during emergence in maize. Consequently, it sheds light on the responses of young maize plants to LT environments, specifically through changes in their transcriptomic expression. Insights from this study lay the groundwork for subsequent network analyses in later developmental phases and across diverse maize genotypes, as well as the potential mechanisms underlying the maize low-temperature tolerance/susceptibility. As such, it offers valuable guidance for future research directions in the molecular breeding of chilling-tolerant maize.

## Data Availability

The datasets presented in this study can be found in online repositories. The names of the repository/repositories and accession number(s) can be found below: https://www.ebi.ac.uk/ena, PRJEB80094.
