# Supplementary material for: Maize transcriptome profiling reveals low temperatures affect photosynthesis during the emergence stage
Source: Front Plant Sci. 2025 Jan 28;16:1527447. doi: 10.3389/fpls.2025.1527447 (PMC11810925; doi:10.3389/fpls.2025.1527447)
Supplement: Supplementary file 1 [file Image1.pdf]

Supplementary Figure S1 Validation of selected differentially expressed mRNAs using qRT-PCR

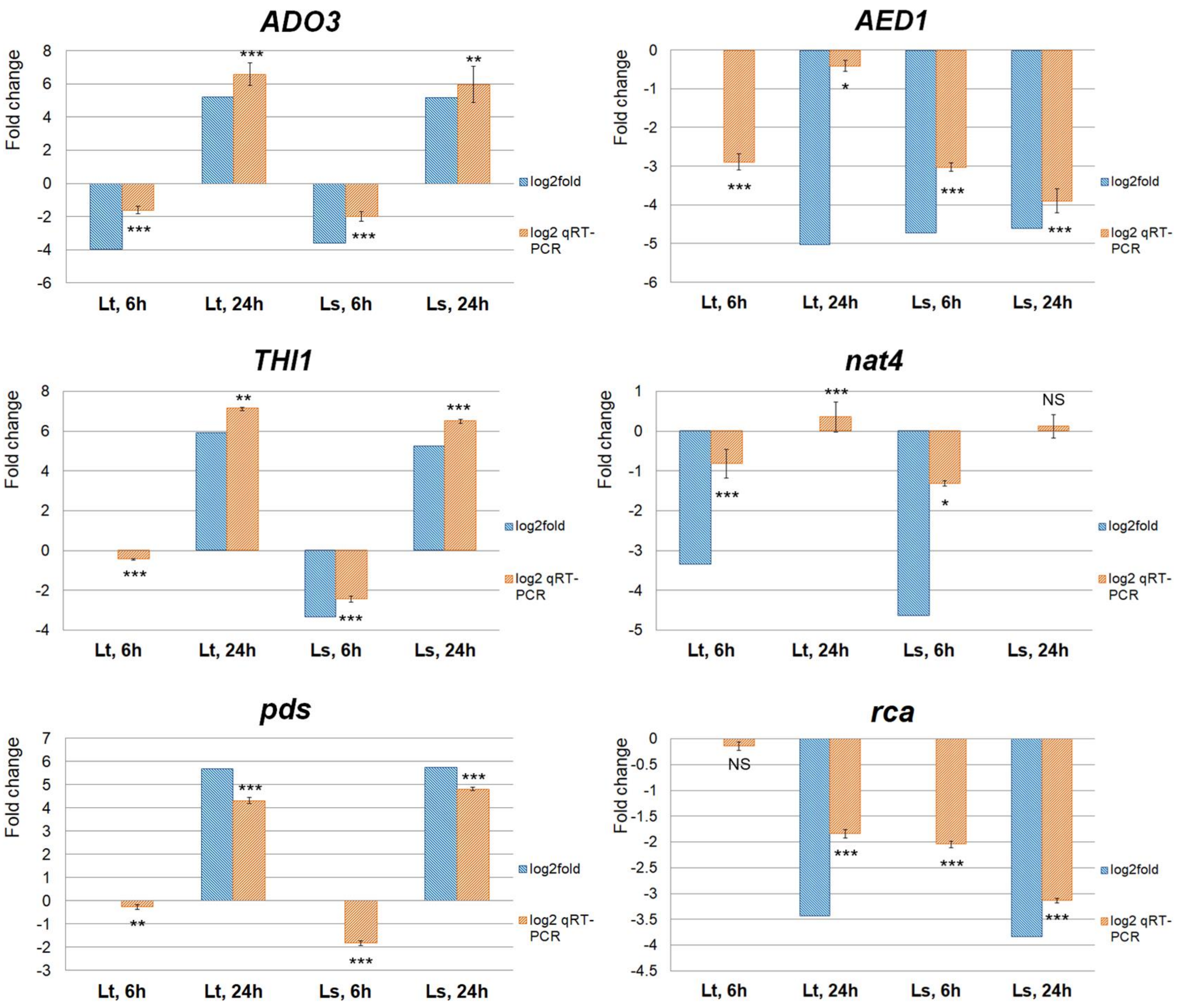

Supplementary Figure 1. Validation of the selected mRNAs (*ADO3, THI1, AED1, nat4, pds, rca*),using qRT-PCR.The expression patterns obtained from the next-generation sequencing are shown as the log2 fold changes between the control and treated samples in L_T and L_S after 6h and 24h. qRT-PCR expression patterns are shown as log2($2^{-\Delta\Delta Ct}$ values) obtained from the ΔΔCt values from control and treated samples in LT and LS after 6h and 24h. The significance of the difference between the control and treatment of each parameter was determined by the t-test and is shown as *** ($p < 0.001$), ** ($p < 0.01$), * ($p < 0.05$), and NS

(statistically not significant at $p < 0.05$).
