# Supplementary material for: Maize transcriptome profiling reveals low temperatures affect photosynthesis during the emergence stage
Source: Front Plant Sci. 2025 Jan 28;16:1527447. doi: 10.3389/fpls.2025.1527447 (PMC11810925; doi:10.3389/fpls.2025.1527447)
Supplement: Supplementary file 2 [file Image2.pdf]

## Supplementary Figure S2 Validation of selected differentially expressed lncRNAs using qRT-PCR

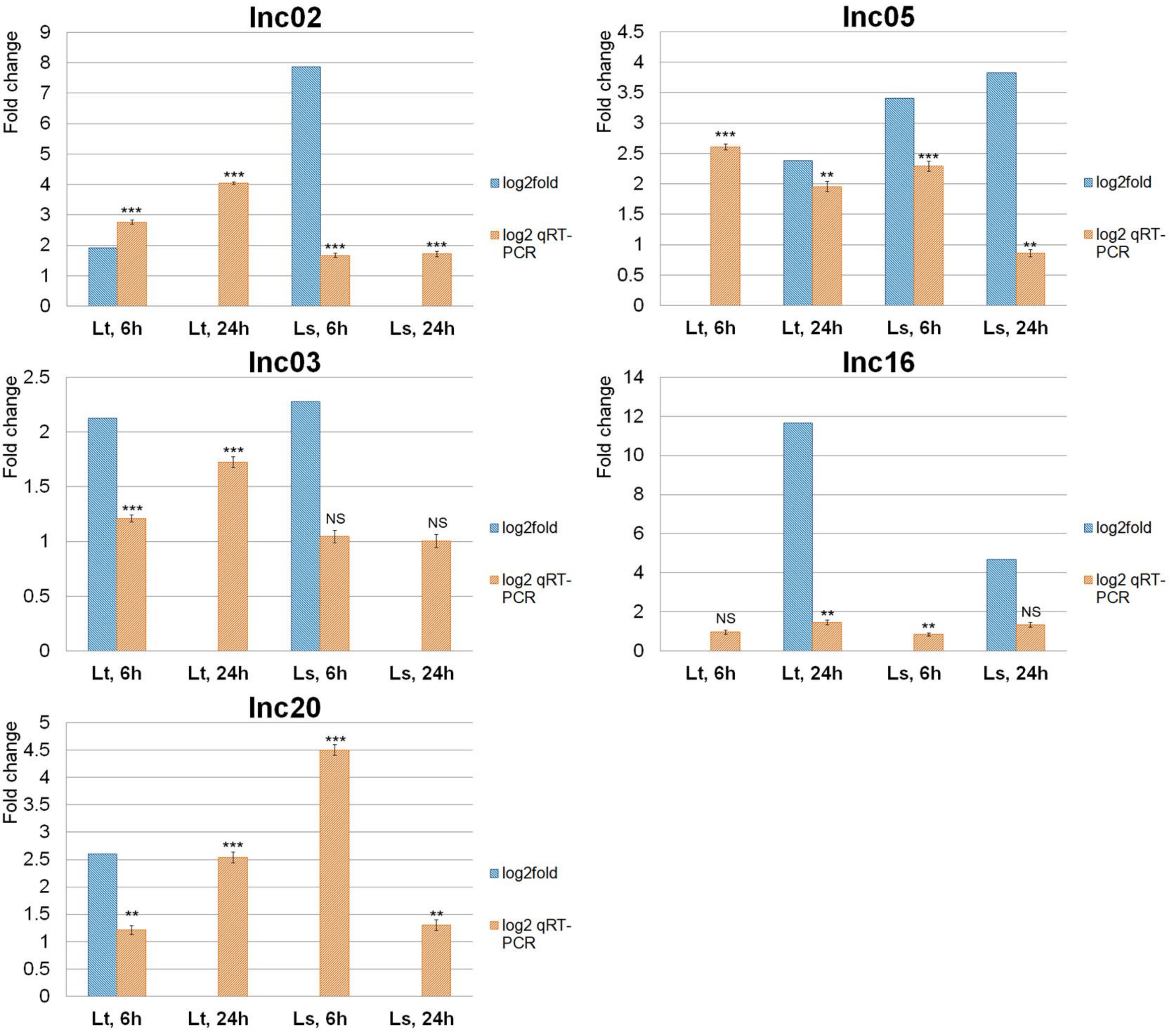


**Supplementary Figure 2.** Validation of the selected lncRNAs (XLOC_012565 - lnc02, XLOC_000175 - lnc03, XLOC_001043 - lnc05, XLOC_006714 - lnc16, XLOC_016783 - lnc20), using qRT-PCR. The expression patterns obtained from the next-generation sequencing are shown as the log2 fold changes between the control and treated samples in LT and LS after 6h and 24h. qRT-PCR expression patterns are shown as log2($2^{-\Delta\Delta Ct}$) values obtained from the ΔΔCt values from control and treated samples in $L_T$ and $L_S$ after 6h and 24h. The significance of the difference between the control and treatment of each parameter was determined by the t-test and is shown as *** ($p < 0.001$), ** ($p < 0.01$), * ($p < 0.05$), and NS (statistically not significant at $p > 0.05$).
