# Supplementary material for: Maize transcriptome profiling reveals low temperatures affect photosynthesis during the emergence stage
Source: Front Plant Sci. 2025 Jan 28;16:1527447. doi: 10.3389/fpls.2025.1527447 (PMC11810925; doi:10.3389/fpls.2025.1527447)
Supplement: Supplementary file 3 [file Image3.pdf]

Supplementary Figure S3 Differentially expressed genes involved in photodamage limitation

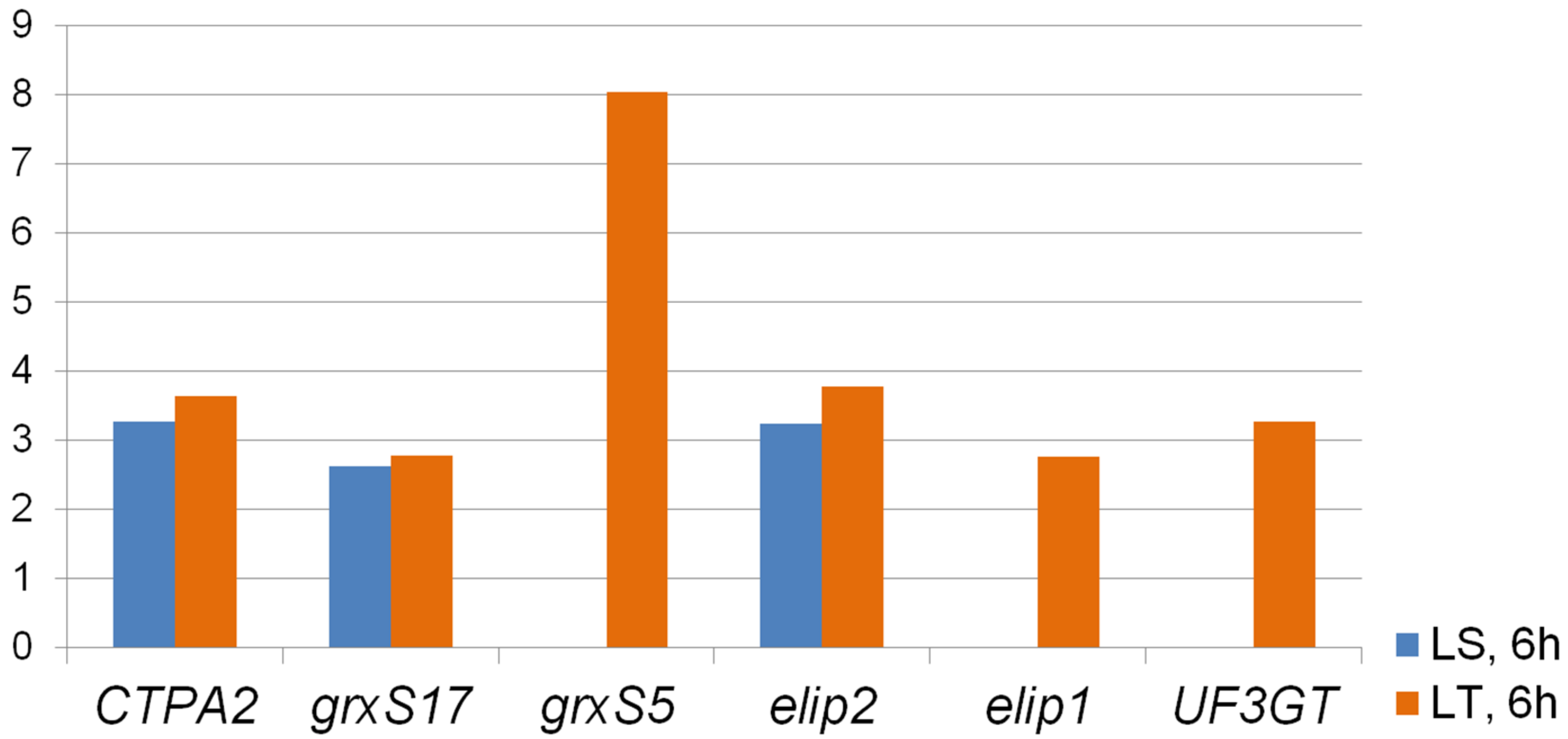

Supplementary Figure S3. Differentially expressed genes involved in photodamage limitation. Differential expression (DE) is shown as the log2 fold change between the control and treated samples in LT and LS after 6h (y-axis). LT, 6h is shown in orange, and LS, 6h is in blue. The DE genes are presented on the x-axis: carboxyl-terminal-processing peptidase 2 (*CTPA2*), glutaredoxin S17 *(grxS17*) and S5 (*grxS5*), early light inducible protein 2 (*elip2*) and 1 *(elip1*), anthocyanidin 3-O-glucosyltransferase (*UF3GT*).
